# Potential Impact of Prosthetic Biomaterials on the Periodontium: A Comprehensive Review

**DOI:** 10.3390/molecules28031075

**Published:** 2023-01-20

**Authors:** Mario Alberto Alarcón-Sánchez, Artak Heboyan, Gustavo Vicentis de Oliveira Fernandes, Natividad Castro-Alarcón, Norma Samanta Romero-Castro

**Affiliations:** 1 Department of Microbiology, Faculty of Chemical and Biological Sciences, Autonomous University of Guerrero, Chilpancingo 39090, Guerrero, Mexico; 2 Department of Prosthodontics, Faculty of Stomatology, Yerevan State Medical University after Mkhitar Heratsi, Str. Koryun 2, Yerevan 0025, Armenia; 3 Department of Periodontics and Oral Medicine, University of Michigan School of Dentistry, Ann Arbor, MI 48109, USA; 4 Department of Implantology and Oral Rehabilitation, Faculty of Dentistry, Autonomous University of Guerrero, Acapulco 39610, Guerrero, Mexico

**Keywords:** prosthetic biomaterials, fixed prosthesis, periodontal health, subgingival microbiota, periodontal biomarkers and gingival crevicular fluid

## Abstract

The success of a prosthetic treatment is closely related to the periodontal health of the individual. The aim of this article was to review and present the importance of prosthetic restorative materials on the condition of the periodontium, the changes that occur in the composition of the subgingival microbiota and the levels of inflammatory markers in gingival crevicular fluid. Articles on the influence of different prosthetic restorative materials on subgingival microbiota and proinflammatory cytokines were searched for using the keywords “prosthetic biomaterials”, “fixed prosthesis”, “periodontal health”, “subgingival microbiota”, “periodontal biomarkers” and “gingival crevicular fluid” in PubMed/Medline, Science Direct, Scopus and Google Scholar. The type of material used for prosthesis fabrication together with poor marginal and internal fit can result in changes in the composition of the subgingival microbiota, as well as increased accumulation and retention of dentobacterial plaque, thus favoring the development of periodontal disease and prosthetic treatment failure. Biological markers have helped to understand the inflammatory response of different prosthetic materials on periodontal tissues with the main purpose of improving their clinical application in patients who need them. Metal-free ceramic prostheses induce a lower inflammatory response regardless of the fabrication method; however, the use of CAD/CAM systems is recommended for their fabrication. In addition, it is presumed that metal-ceramic prostheses cause changes in the composition of the subgingival microbiota producing a more dysbiotic biofilm with a higher prevalence of periodontopathogenic bacteria, which may further favor periodontal deterioration.

## 1. Introduction

The periodontium constitutes the tissues that support the teeth, it is made up of two soft tissues (which are the gingiva and periodontal ligament) and two hard tissues (which are the root cementum and alveolar bone) [1]. It is now widely accepted that periodontal disease (PD) is a multifactorial pathological entity induced by polymicrobial dysbiosis and host-mediated inflammation [2,3]. Key components in the pathophysiology of PD and its associated clinical features include gingival inflammation, periodontal ligament destruction, bone loss, bacterial colonization and invasion, increased numbers of polymorphonuclear (PMN) and epithelial cells, increased volume and decreased pH of the gingival crevicular fluid (GCF), as well as increased periodontal and gingival indices [4,5,6]. PD has a high prevalence worldwide, which is estimated to be 30–50% [7,8]. Currently, the most recent classification of PD is based on the severity (stages I–IV) and progression (grade A–C) of the disease [9]; however, for practical purposes, we can divide it into gingivitis, which refers to inflammation of the gums, and periodontitis, where in addition to inflammation there is also the destruction of periodontal tissues [10].

One of the factors that precisely leads to the development of periodontal disease is the use of poorly fabricated prosthetic restorations, with a poor marginal and internal fit greater than 120 μm [4]. In this way, and due to a greater marginal discrepancy, the cement forms a thicker layer and comes into contact with the oral cavity environment, which causes the dissolution of the cement and leads to increased accumulation and retention of bacteria in the area causing irreversible damage to the periodontal and pulpal tissues if not detected in time [11,12].

In addition, prosthetic restorative biomaterials can affect the formation of biofilms mainly because of their rough and irregular surfaces creating a series of niches in which microorganisms are protected from tooth brushing, muscular action and salivary flow favoring bacterial colonization and thus in turn the generation of an immunological response by the patient [13]. For this reason, the choice of a restorative material is an important part for the success of a prosthetic treatment in patients who need it, with the main objective of restoring function and esthetics, without leaving aside the biocompatibility and periodontal health that firmly accompany this process, ensuring greater durability of the restoration [14].

Nowadays, ceramics have become increasingly popular as prosthetic restorative materials due to the previously mentioned characteristics [15]; in fact, the most frequently used ceramic restorations in the dental area are porcelain-fused-to-metal crowns [16]; however, the use of metal-free ceramic restorations such as two-layer and single-layer (monolithic) zirconia prostheses has also increased in recent years, becoming a very promising alternative [17]. One of the main disadvantages in the use of metal-ceramic restorations is that the metal alloy can produce allergic reactions in some patients [18,19] and changes in the subgingival microbiota [20]. Finally, it has been observed through studies that this type of restoration fabricated by the conventional method has a lower marginal fit compared to zirconia restorations fabricated by the computer-aided design/computer-aided manufacturing (CAD/CAM) method, a system qualified as an effective and safe fabrication technology for producing fixed prosthetic restorations [21,22], which favors the accumulation of dentobacterial plaque, the formation of dental caries and the development of periodontal and pulp disease [4].

The diagnosis of periodontal disease is mainly performed by evaluating certain clinical and radiographic parameters that allow the dentist to determine the periodontal condition of the patient [23]. In addition, during the last three decades, research has advanced significantly in the field of oral biomarkers, and a wide variety of them have been detected in different fluids such as saliva and GCF, with the main purpose of improving early detection rates of periodontal disease and, in the future, replacing the form of diagnosis to a less invasive and more practical tool [24,25].

The purpose of this review was to provide a detailed summary of the effects of prosthetic restorative biomaterials on the periodontium, changes in subgingival microbiota and levels of biomarkers of inflammation in gingival crevicular fluid.

## 2. Biomaterials Used in Fixed Dental Prosthesis

**Figure 1:**
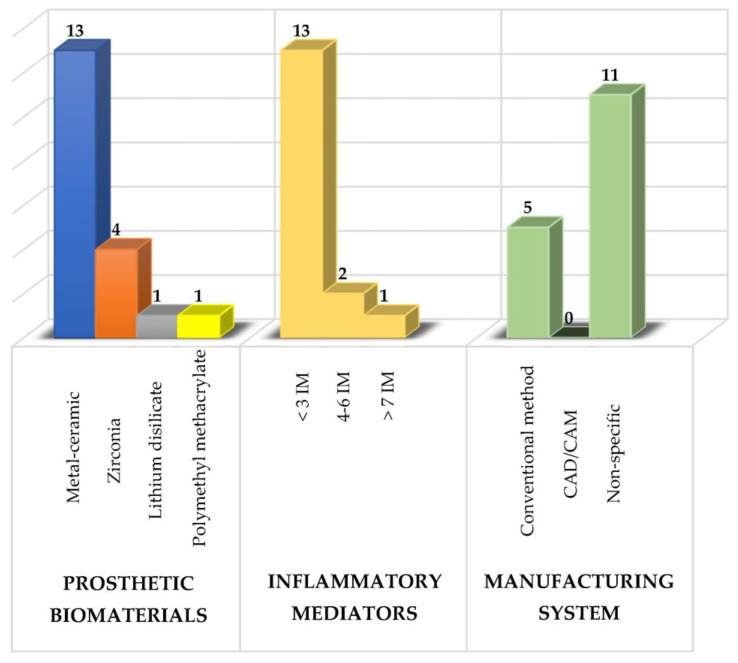
Summary of the study characteristics used in the review. IM: inflammatory mediators.

Several prosthetic materials are used for the fabrication of fixed dental prostheses (as shown in Figure 1), including metal-ceramic and metal-free ceramics such as zirconium oxide and lithium disilicate [26,27,28,29,30,31,32,33,34,35,36,37,38,39,40,41], as well as polymeric materials such as polymethylmethacrylate (PMMA), with the latter mainly used for provisional purposes [28,42].

Of these prosthetic biomaterials, researchers have most frequently analyzed the use of metal-ceramic prostheses followed by zirconium prostheses with the main purpose of knowing their effects on the composition of the subgingival microbiota, the levels of various inflammatory mediators and the periodontal condition to determine which type of prosthetic biomaterial induces a lower inflammatory response and thus to have a therapeutic alternative that maintains the patient’s periodontal health [4,6].

Metal-ceramic prostheses are composed of a metal coping that supports the overlying ceramic. They are characterized because they are ideal where there is little tooth structure and are more economical compared to metal-free ceramics [43]. In relation to the use of this type of prosthesis, twenty-one different inflammatory mediators have been analyzed [26,29,30,32,33,34,35,36,37,38,39,40,41], and currently, it is very well documented that metal ceramic prostheses increase bacterial levels; therefore, there is a greater production of proinflammatory cytokines which leads to the destruction of the supporting tissues of the teeth [20,26]. This is partly due to the fact that the bacteria in the biofilm lower the pH by producing acidic substances that dissolve the surface oxides of the dental alloys, reducing the resistance to corrosion and, therefore, generating rough and irregular surfaces that favor a greater accumulation and retention of bacteria in the area [44].

On the other hand, the use of metal-free ceramic prostheses, mainly zirconia restorations [45], has increased in recent years, becoming a very promising alternative [17]. This type of restoration is very durable, require a minimally invasive preparation, which allows a greater preservation of the dental tissue, a high resistance to bending and fracture, as well as their translucency property to be retained; however, they are more expensive than metal-ceramic prostheses [46]. As a consequence of the use of this type of biomaterials, less biofilm formation has been found and therefore a more accentuated decrease in the levels of proinflammatory cytokines [26,28,29,31,47].

## 3. Periodontal Health in Patients with Fixed Dental Prosthesis

An ideal prosthetic treatment should not only be limited in restoring function and esthetics in the patient, but also achieve a healthy relationship with the periodontal tissues [48,49,50,51]. Thus, there are some factors that influence periodontal health such as cervical emergence profile, periodontal phenotype, biological thickness, materials and fabrication method of the restoration, prosthetic margin location, prepared tooth finish line, cementing materials and marginal and internal fit [10,12,52].

The cervical emergence profile or apical third design of a restoration is defined as the contour of the tooth and crown as they cross the soft tissues and rise toward the interproximal contact zone and the height of the facial and lingual contour [53]. On the other hand, the periodontal phenotype corresponds to the gingival thickness and width of the keratinized tissue (gingival phenotype) and bone morphotype [54]. This can be divided into thick and thin phenotypes. The thin periodontal phenotype represents a small proportion of cases [55]; however, it is considered a risk factor for additional bone loss [56], is associated with gingival recession [57] and also more prone to increasing the severity of peri-implantitis [58]. Having knowledge of the different periodontal phenotypes helps to minimize tissue damage and provides better results both in preparing the tooth for prosthetic placement and in gum recession. The biological thickness is defined as the dentogingival junction constituted by the junctional epithelium and the insertion of the supraalveolar connective tissue (2.04 mm). This space must be respected in order to maintain and protect periodontal health. Prostheses made and placed in an iatrogenic manner, i.e., violating the biological thickness, predispose an individual to the development of subgingival caries and result in an inflammatory process, which ultimately leads to the destruction of periodontal tissue [59,60].

In relation to biomaterials and the method of fabrication of prosthetic restorations, it has been observed that patients with zirconia prostheses obtain better results in terms of periodontal health, reduction of inflammation and maintenance of oral hygiene compared to metal-ceramic prostheses [57]. On the other hand, the cytomorphometric analysis of the periodontium before and after the insertion of fixed Cr-Co metal-ceramic prostheses fabricated by the conventional method and the CAD/CAM system, as well as the use of zirconia prostheses fabricated by the latter technique has shown an increase in the oral epithelial cell count and a decrease in the PMN count. Moreover, the cytological method is an informative test that allows us to identify etiological risk factors of the periodontitis, since it reveals the dynamics of the disease during its progression in prosthetic treatment [61].

The location of the prosthetic margin in relation to the completion line can be subgingival (below the gingival margin), juxtagingival (at the level of the gingival margin) or supragingival (above the gingival margin) [62]. The finishing line of a dental preparation is defined as the junction of the prepared and unprepared tooth structure with the margin of the restoration [49], so there are three types: horizontal, including straight shoulder, beveled shoulder, curved and sloped chamfer; vertical, including knife-edge preparation; and the preparation without a finishing line (BOPT). In fact, it has been shown that anterior teeth treated with the biologically oriented preparation technique (BOPT) present better plaque indices, stable probing depth, greater gingival thickness and stable gingival margins. In addition, prosthetic treatment using this technique has a positive impact on patient satisfaction, and based on these results, the authors highly recommend this technique, especially in cases of retreatment with prosthetic crowns [14]. The marginal and internal fit corresponds to the space between the margin of the restoration and the finishing line of the prepared tooth [11]. It is accepted that this space should not be >120 μm. In this way, with a thorough evaluation of the periodontium, an individualized and precise periodontal treatment can be proposed, since each case is different. It is also very important to motivate the patient to place greater emphasis on brushing and plaque control. In addition, maintenance appointments should be taken into account to avoid possible negative effects on the periodontium associated with the use of prosthetic restorations [63].

## 4. Changes in the Composition of the Subgingival Microbiota Related to the Use of Fixed Dental Prosthesis

The subgingival microbiota corresponds to diverse microbial communities (bacteria, archaea, fungi and viruses) that live attached to the root surface of teeth or dental implants with their outer surface in contact with the gingival tissue [64,65]. Bacteria are the most abundant component, and it is estimated that there are approximately 500 species that live in a state of eubiosis with the host [66,67,68]. However, the microbiota can undergo substantial changes as a result of various factors (unbalanced diet, smoking and poor oral hygiene) [69,70] that disrupt bacterial homeostasis and lead to a state of dysbiosis, where one or more types of periodontopathogenic bacteria proliferate, at least temporarily taking over the immune system, as happens in gingivitis and periodontitis [71,72]. A representation showing the associations between bacterial species colonizing the gingival sulcus is that of bacterial complexes in ecological equilibrium [67], where *Porphyromonas gingivalis, Tannerella forsythia* and *Treponema denticola*, which are the most periodontopathogenic bacteria constituting the red complex, have been detected in higher proportions in periodontitis conditions compared to healthy subjects [73,74,75,76] and even in patients with periodontitis and other systemic diseases [77].

On the other hand, in addition to natural teeth, dental implants and dentures are substrates for biofilm formation [78,79]. In relation to prosthetic restorations, it has been observed that the formation of biofilms on different types of dental ceramics is highly dependent on the genus and species of the microorganism [80]. In fact, the ability of microorganisms to adhere to prosthetic restorative materials has been mainly associated with the chemical composition of the biomaterial, the surface roughness, the surface free energy, its irregular topography and the release of metal ions, which could contribute to biofilm growth and generate a pathological process [44]. The most recent studies regarding the influence of metal-ceramic prosthetic restorations on the composition of the subgingival microbiota indicate a higher proportion of orange and red complex bacteria associated with a higher accumulation of dentobacterial plaque and bleeding on probing which is why they could be more vulnerable to future periodontal deterioration in case of a sudden change in the host immune response [20,36,81,82]. Additionally, the subgingival microbiota around single tooth implants has been evaluated and compared with natural teeth, finding a higher proportion of *Klebsiella pneumonie*, *Pseudomonas aeruginosa* and *Streptococcus* species compared with their controls, taking into account that the first two species are infrequently found in the oral cavity and are related to cases of periodontitis and peri-implantitis [83]. On the other hand, it has also been demonstrated that, in CAD/CAM fabricated zirconia prosthetic restorations, bacterial levels are more compatible with periodontal health, producing a more pronounced change towards clinical recovery in these patients, since zirconia is a highly biocompatible material with periodontal tissues, has less negative effects on gingival margins and also greatly inhibits biofilm formation producing a more subdued inflammatory response compared to metal-ceramic prostheses fabricated mainly by the conventional method [47].

With this, what is expected in the future is to be able to implement therapies that help to control and reduce the formation of biofilm around prosthetic restorations. In fact, in addition to conventional treatment (scaling and root planning), the use of photoactivation antimicrobial therapy, which combines the activation of a photosensitizer with a light source in the presence of oxygen, producing free radicals that generate damage to bacteria, has had very important microbiological and clinical results, especially in cases of patients with severe periodontitis and fixed dental prosthesis. For this reason, it is expected that this therapy will help to solve the problems and difficulties faced by conventional antimicrobial therapy and can function as a complement to conventional mechanical treatments [84,85].

## 5. Oral Biomarkers

In the oral cavity, the biological means to detect biomarkers in relation to periodontal disease are GCF, saliva, mouth rinses, dentobacterial plaque (supragingival and subgingival) and tissue biopsies [86,87]. The GCF is composed of a range of molecules from blood, host tissues and biofilm, including inflammatory mediators such as cytokines and chemokines, leukocytes, enzymes, organic and inorganic ions, tissue degradation products and other proteins [88]. Under normal conditions, the gingival sulcus contains a minimal amount of GCF; however, during inflammation of the periodontium, this fluid travels from the capillary structure into the inflamed connective tissue producing such exudate. GCF provides information on the volume of fluid in gingival inflammation to assess health and disease status [89]. GCF collection methods include intracrevicular lavage technique, microcapillary technique and absorption technique through the use of paper strips being the easiest and most accurate method for obtaining such exudate [90]. The enzyme-linked immunosorbent assay (ELISA) is the method preferred by researchers for the analysis of GCF samples because it has been very efficient in the field of periodontal disease diagnosis [91,92].

### 5.1. Classification of Oral Biomarkers

**Figure 2:**
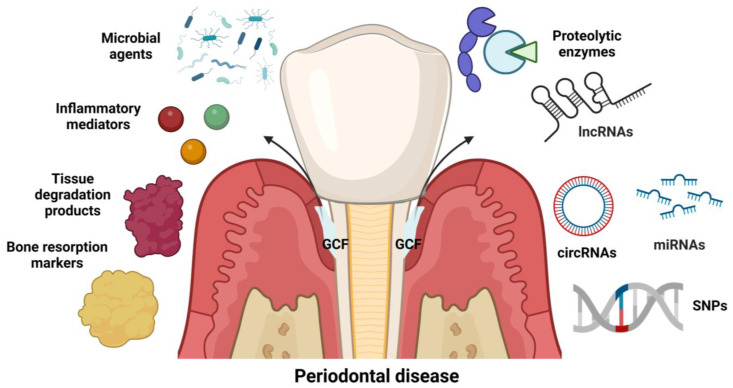
Classification of oral biomarkers according to biological type. IncRNAs: long non-coding RNAs; circRNAs: circular RNAs; miRNAs: microRNAs; SNPs: single nucleotide polymorphisms; GCF: gingival crevicular fluid. Created with www.biorender.com (accessed on 9 December 2022).

In the area of periodontics, biomarkers can be classified into two types. The first is according to the diagnostic information requested, such as predictive, initial diagnostic, prognostic and maintenance biomarkers [92]. The second one is in the function of the biological type, where it is currently known that there are different types of inflammatory mediators, tissue degradation products, bone resorption markers, microbial agents, proteolytic enzymes [91,93], non-coding RNAs [93,94,95,96] and single nucleotide polymorphisms (SNPs) [97] (Figure 2).

### 5.2. Biomarkers of Inflammation

Nowadays, the field of research regarding oral markers, especially inflammatory mediators such as cytokines and chemokines, has advanced significantly [91], and a wide variety of them have been used in the diagnosis of patients with periodontitis, as is the case with TNF-α [98], CXCL10, IL-6, CXCL13, IL-8, IFN-γ, IL-10 [99], IL-18 [100], IL-21 [101] and the IL-23/IL-17 axis [102]. However, other molecules such as azurocidin (AZU) and fractalkine (CX3CL1) have recently been proposed as potential markers of periodontal disease [103,104].

#### Biomarkers of Inflammation in Relationship to the Use of Fixed Dental Prosthesis

A variety of inflammatory mediators such as IL-1α, TNF-α, IL-1β, IL-6, MIP-1, IL-8, IL-1ra, CRP, PGE2 and IgG have been used to gain insight into the inflammatory response as a consequence of the use of different prosthetic restorative materials [27,28,29,30,31,32,33,34,36,37,38,39,40,41] and even some enzymes such as resistin, aspartate aminotransferase, alkaline phosphatase and matrix metalloproteases (MMPs), mainly MMP-2, MMP-8, aMMP-8 and MMP-9, which have been implicated in the destruction of periodontal tissues [26,35] (Table 1).

##### TNF-α

Tumor necrosis factor alpha (TNF-α) is a pleiotropic cytokine with proinflammatory functions [105], which is part of the tumor necrosis factor (TNF) superfamily [106]. It is encoded by the *TNFA* gene located on chromosome 6p21 [107] and was first purified and characterized by Aggarwal et al. in 1985 [108]. It is generally produced by macrophages [109], T cells and NK cells [110]; however, it can also be secreted by fibroblasts present in the periodontal ligament, gingival keratinocytes and osteoblasts [111]. It is first synthesized as a type II transmembrane protein on the cell surface of 27 kDa and consists of 233 amino acids, which undergo proteolytic cleavage between alanine 76 and valine 77 residues by a matrix metalloproteinase named the TNF-α, converting enzyme (TACE), releasing the soluble TNF-α homotrimer of 17 kDa, and consisting of 157 amino acids [112]. Both forms of the protein bind and interact with two types of receptors—tumor necrosis factor receptor type 1 (TNFR1) and type 2 (TNFR2)—promoting the activation of multiple signaling pathways involved mainly in cell apoptosis and necrosis, as well as in cell migration and survival [113].

Regarding the response of the periodontium after the placement of different prosthetic restorative materials [114,115,116,117,118,119], ceramic lumineers are currently considered the most conservative indirect restorations among minimally invasive esthetic treatments. However, despite having a very thin thickness compared to conventional veneers, lumineers are overcontoured because the tooth wear is nil, which may contribute to increased plaque accumulation and retention compromising the individual’s periodontal health. Initially, treatment with ceramic lumineers has shown a transient increase in TNF-α and IL-6 levels indicating the onset of gingival inflammation; however, after a few weeks have elapsed, the levels of these cytokines normalize, suggesting that their clinical application for esthetic rehabilitation is a viable option with minimal risks of compromising periodontal health [27]. Taking into account that a healthy and stable periodontal tissue is an important factor for prosthetic restoration, the effects and periodontal condition of teeth with chromium-cobalt (Cr-Co) alloy and zirconium dioxide-based metal-porcelain prostheses have also been compared. In this case, it has been shown that TNF-α and C-reactive protein (CRP) levels decrease in both groups one day after restoration, with significantly lower levels in patients with zirconia restorations, indicating that the inhibition effect of metal-free ceramic prostheses on inflammation is more prominent [26]. Likewise, it has also been shown that patients with periodontitis and with Cr-Co or nickel-chromium (Ni-Cr)-based metal restorations show a more pronounced inflammatory reaction with increased levels of MMP-8, IL-1β, IL-6 and TNF-α compared to patients with periodontal involvement but without prosthetic restorations demonstrating once again that metal ceramic prosthetic restorations could induce a more marked deterioration of periodontal tissues [35].

##### IL-1β

Interleukin 1 beta (IL-1β) is a cytokine of a proinflammatory nature which belongs to the interleukin 1 family. It is encoded by the *IL1B* gene and was initially discovered as the major endogenous pyrogen. It is produced by monocytes/macrophages, dendritic cells [120], gingival fibroblasts, periodontal ligament cells and osteoblasts. Its production has been increased and altered in multiple inflammatory disorders, mainly rheumatoid arthritis, cryopyrin-associated periodic syndrome (CASP), gout, type II diabetes mellitus, as well as in periodontitis [121,122]. Its secretion is unique, because first the gene is transcribed and translated as an inactive precursor, 35 kDa pro IL-1β; then it is cleaved between aspartic acid 116 and alanine 117 to generate its active 17 kDa form by the action of caspase-1 (CASP1) [123,124]. After IL-1β secretion, the expression of collagenolytic enzymes, such as matrix metalloproteinases (MMPs) (mainly MMP-8, MMP-9 and MMP-13), is increased [125,126], which contributes to the degradation of the extracellular matrix. This, in turn, also increases the synthesis of PGE2 in fibroblasts, as well as the expression of CX3CL1, which modulates the migration of osteoclast precursors that then lead to bone resorption and tissue destruction [121,122].

To date, IL-1β is the most extensively studied cytokine in relation to the effects or influence that different prosthetic materials may have on the periodontium, including other temporary restorative materials such as dental cements and nanohybrid composites [127]. In fact, it has been observed that patients with zirconia-based fixed dental prostheses show a decrease in IL-1β levels compared to those with porcelain metal prostheses and temporary prosthetic materials, such as PMMA, where there is a very slow decrease in this cytokine indicating a more inflammatory state [28,29,32,40,41]. Additionally, the inflammatory reaction between metal-free ceramic prosthetic restorations such as lithium disilicate and zirconia prostheses has been quantified by measuring the concentration of inflammation indicators in GCF. Based on the results, there is no significant difference between the levels of this cytokine, indicating that both metal-free prosthetic materials are biocompatible with periodontal tissues [31]. On the other hand, in relation to the location of the prosthetic margins with the tooth preparation finishing line, it has been shown that, in sites where the crown margins are located above the gingival margin, IL-1β levels are lower compared to juxtagingival and subgingival margins, thus demonstrating that supragingival restorations seem to be more suitable for promoting periodontal health compared to the other marginal finishing lines [37].

##### IL-6

Interleukin 6 (IL-6) is a soluble mediator of 212 amino acids, with a molecular weight of 21 to 26 kDa [128]. It belongs to the IL-6 family together with seven other cytokines [129], is encoded by the *IL6* gene located on chromosome 7p21, and has been given several names due to its multiple biological activities [130,131].

It is synthesized in response to infections or trauma [129], mainly by macrophages, neutrophils, keratinocytes, gingival fibroblasts and endothelial cells [132,133], meaning that under normal conditions, IL-6 levels are very low; however, these can increase in inflammatory states [130], as seen in patients with periodontitis and other systemic diseases [134,135].

IL-6 has both proinflammatory and anti-inflammatory functions. The proinflammatory activities are modulated by signal transmission through sIL-6R [130] in a process termed trans-signaling [136] and include activities such as inflammatory cell recruitment and inhibition of regulatory T cell differentiation. In contrast, its anti-inflammatory functions are mainly executed through membrane-bound IL-6R [130], termed classical signaling [136], and include biological processes such as differentiation of B lymphocytes with consequent antibody production, activation and differentiation of T lymphocytes, induction of angiogenesis (vascular permeability and osteoclastic differentiation), as well as increased production of acute phase proteins [129,137].

As mentioned, some dental alloys cause inflammation in the periodontium, and IL-6 is no exception. The influence of some pure metals and ceramics on cell viability and the synthesis of this cytokine in human gingival fibroblasts and keratinocytes has been recorded, and it has been found that the vitality of these cells is reduced after exposure to metals such as copper, cobalt, zinc and nickel. Moreover, there is an increase in the levels of this cytokine with the previously mentioned metals in comparison with ceramics, which is why it is suggested that metal ions are involved in proinflammatory activity [138]. Therefore, ceramic particles induce a lower immune response compared to Cr-Co particles [139]. In this sense, the level of gingival irritation and cytotoxicity caused by the presence of porcelain crowns with Ni-Cr alloy has also been explored in order to analyze the levels of IL-6 in GCF in different periods. This has demonstrated that IL-6 concentration increases significantly at one week, three months and six months later, with this being more evident after the third month and suggesting that IL-6 can be considered an indicator of gingival irritation caused by the release of nickel ions [34]. Likewise, correlations between microbiological and inflammatory parameters and clinical indicators of success/failure on periodontal condition in metal-ceramic prostheses have also been evaluated in order to search for parameters that can be considered predictors of failure. This has demonstrated that subjects with gingivitis and periodontitis present a significant increase in the levels of IL-6 and other cytokines involved in the inflammatory response, such as IL-1β and TNF-α, compared to periodontally healthy subjects. These data confirm that the use of metal-ceramic prostheses could be causative of a local and sustained inflammatory process, which would ultimately translate to greater tissue damage. This risk is possibly associated with microbiological and host factors that predispose an individual to the appearance of periodontal alterations in areas reconstructed with metal-ceramic crowns [36].

##### IL-8

Interleukin 8, also named CXC motif chemokine ligand 8 (CXCL8), is a proinflammatory chemokine that is encoded by the *IL8* gene located on chromosome 4q13-q21. It is a member of the α-subfamily of chemokines characterized by a conserved tripeptide near the N-terminal end, containing cysteines (CXC) [140,141]. It is produced by lymphocytes, monocytes, macrophages, gingival fibroblasts and keratinocytes [134]; this is due to stimulation by periodontopathogenic bacteria such as red complex bacteria at the site of infection [141,142]. IL-8 not only induces effects such as neutrophil adhesion across the vascular endothelium, but also stimulates exocytosis of its granules leading to the release of lysosomal enzymes [141,143] by activating multiple signaling pathways through CXCR1 and CXCR2 receptors [144]. In periodontitis conditions, increased IL-8 gene expression and higher IL-8 protein levels have been observed compared to healthy subjects; therefore, it is another important chemokine of interest in PD [145].

On the other hand, it has been shown that the presence of metal ions in osteoblastic cell culture media induces the production of proinflammatory cytokines such as IL-8, a potent chemoattractant for phagocytes (macrophages and neutrophils), and IL-6, an activator of osteoclasts [146]. In fixed prosthodontics, the effects of prosthetic crown margin placement on IL-1α and IL-8 levels in FCG have been analyzed, and it has been demonstrated that IL-8 levels are higher in samples taken at subgingival and juxtagingival margins compared to supragingival margins. Subgingival and juxtagingival margins elicit a greater inflammatory response along with a worse prognosis for prosthetic restoration compared to subgingival and juxtagingival margins regardless of oral health status. Prosthetic margin placement can influence and affect plaque control and jeopardize the health of the supporting tissues of the teeth; therefore, it is very important to have good treatment planning and consider the selection of the prosthetic margin to avoid further destruction [33]. Finally, IL-6 and IL-8 levels in FCG of fixed partial denture bearing abutment teeth have also been analyzed, and it has been shown that, after non-surgical periodontal treatment, there is a reduction in the amount of IL-8; however, there is a tendency for there to be higher levels of probing depth and periodontal and gingival index in these patients. Therefore, attending maintenance appointments through a regular program of dental prophylaxis is also important to improve periodontal health in patients with fixed prostheses [38].

## 6. Potential Biomarkers of Periodontal Disease

### 6.1. Azurocidin

Azurocidin (AZU), also referred to as heparin-binding protein (HBP) or 37 kDa cationic antimicrobial cationic protein (CAP 37), is a 29 kDa neutrophil-derived protein, which was first identified in 1984 by Shafer et al. It belongs to the serine protease family and possesses 222 amino acid residues [147,148]. It has the characteristic of being proteolytically inactive, and this is due to the substitution of residues histidine 41 by serine and serine 175 by glycine, while the third member of the catalytic triad aspartic acid 89 is conserved [149,150,151].

Polymorphonuclears are generally considered to be the only cells that secrete AZU; however, monocytes can also synthesize it in small amounts [151]. Different cytokines, antigens and enzymes can stimulate the degranulation of neutrophils to secrete AZU. Their release in some cases is dependent on calcium influx; however, different factors also modulate unique pathways to promote their secretion. One of these is the S. aureus-derived phenol-soluble modulin a4 (PSMa4), which binds to formyl peptide receptor 2 (FPR2) on the surface of PMNs to activate the PI3K pathway and induce AZU release [151,152].

When released, it exerts effects as a potent chemoattractant for monocytes and induces vascular leakage and edema formation [151]. In addition, it contributes to bacterial killing by opsonizing bacteria, which facilitates recognition and uptake by phagocytes [148,152]. Integrating these properties, AZU can be characterized as a proinflammatory protein of rapidly migrating neutrophils and as one of the first lines of defense against infection [147]. In relation to PD, the proteomic profile of chronic periodontitis has been identified, and AZU has been proposed as a potential biomarker [153], with evaluated AZU levels in FCG. Significantly higher levels are found in patients with chronic periodontitis compared to periodontally healthy patients, indicating that this protein is also involved in PD pathogenesis [103,154,155].

### 6.2. Fractalkine (CX3CL1) and Its Receptor (CX3CR1)

Fractalkine or chemokine ligand 1 (C-X3-C motif) (CX3CL1) [156], also referred to as neurotactin [157], is the only member of the CX3C chemokine subfamily [158]. It is characterized by the presentation of a cysteine motif -Cys-XXX-Cys- at the N-terminal end [159]. This chemokine is encoded by the *CX3CL1* gene located on chromosome 16q13 and was first discovered by Bazan et al. in 1997 [160]. It is mainly produced by monocytes, endothelial cells and smooth muscle cells [161]; however, gingival fibroblasts [162] and osteoblasts can also secrete it. CX3CL1 is initially synthesized as a polypeptide consisting of 397 amino acids with 5 domains [163]. After cleavage of the signal peptide sequence, the synthesized polypeptide is glycosylated and then incorporated into the cell membrane constituting a type I transmembrane glycoprotein of 373 amino acids and with a molecular weight of 100 kDa [157,164]. The membrane-bound form has an important function as a cell adhesion molecule and can be induced by proinflammatory cytokines such as interferon-γ (IFN- γ), TNF-α [164] and interleukin 1-β [121] as well as virulence factors such as LPS [165]. This function results from the interaction between the functional chemokine domain present in the extracellular region of the protein with the N-terminal end of the fractalkine receptor (CX3CR1) [163], which is expressed by NK cells, cytotoxic T lymphocytes, monocytes/macrophages, osteoclasts and gingival fibroblasts [162]. On the other hand, CX3CL1 can also be present in its soluble form, which is the result of proteolysis thanks to the action of different enzymes such as TNF-α-converting enzyme/disintegrin-like metalloproteinase 17 (TACE/ADAM17) and ADAM 10 (ADAM10) [166,167]. These cleave the transmembrane hydrophobic region, generating a protein consisting of 317 amino acids that acts as a chemoattractant molecule for leukocytes [164,168]. Thus, both forms of the protein play a very important role in the innate and adaptive immune response by promoting the invasion and selective accumulation of immune cells to the site of injury.

Biological means for detection of this chemokine include FCG, saliva and tissue biopsies, and it has even been detected at the systemic level by collecting a blood serum sample [169]. In relation to PD, it has been shown that levels of fractalkine/CX3CL1, CX3CR1 and IL-1β in FCG are increased in patients with periodontitis compared to periodontally healthy patients [104]. Additionally, as a very close link to rheumatoid arthritis (RA), Yilmaz et al. [169] and Panezai et al. [170] analyzed CX3CL1 levels in patients with periodontitis and RA, finding a statistically significant increase in their levels compared to their control groups represented by systemically and periodontally healthy patients. Additionally, in relation to prosthetic restorations, the influence of titanium implants and long-standing amalgam restorations on the levels of L-Kyn/L-Trp and chemokines such as CX3CL1 and MCP-1 has been evaluated. Thus, it has been shown that CX3CL1 levels are significantly higher in patients with titanium implants and dental amalgam restorations compared to patients with only long-standing dental amalgam restorations [171]. Finally, the immunoexpression of CX3CL1 and its receptor (CX3CR1) has also been evaluated in periodontal tissues with inflammatory infiltrate, demonstrating that leukocytes in diseased periodontal tissue express CX3CR1, while CX3CL1 is strongly expressed in endothelial cells of diseased periodontal tissues [172]. These studies suggest that the Fractalkine-CX3CL1/CX3CR1 axis may have an important role in the development of periodontitis because it may be associated with mechanisms that regulate inflammation, especially the migration of specific leukocytes to inflamed periodontal tissue.

## 7. Strategies for the Resolution of Inflammation Caused by the Use of Different Prosthetic Biomaterials

Highlighting one of the objectives of the present review, which is to show the molecular and immunological aspects of the influence of the prosthetic materials most commonly used for the fabrication of a fixed dental prosthesis and their response to the periodontium, we can guide the general dentist and the specialist in prosthodontics and oral implantology on decision making in the clinic and highlight the following points as strategies for improving the quality and harmony between dental prostheses and the tissues that support them:

The fabrication of a fixed dental prosthesis by a CAD/CAM system, which improves marginal and internal fit, greatly inhibiting biofilm deposition [57].

The use of metal-free ceramic prosthetic restorations, which decrease the production of proinflammatory cytokines and markers of destruction in comparison with metal ceramic prostheses, which would favor a faster recovery of the tissues [26,27,28,29,31].

To improve the cementation technique and to avoid an excess of cementation that could damage the tissues [12].

Continuous education and motivation of the patient to be more meticulous in their oral hygiene, mainly in their dental prosthesis [173].

## 8. Future Perspectives

This review discussed the importance of prosthetic restorative materials on the state of the periodontium, as well as the changes that occur in the composition of the subgingival microbiota and the levels of inflammatory markers in the gingival crevicular fluid. Today, new materials are being sought that exert less of an inflammatory response in the tissues, while still meeting the esthetic and strength requirements, which are important for restoring the oral cavity to a good level of health, esthetics and function [14,174,175,176].

We can mention that some prosthetic restoration materials, despite having many years of use, such as metal-ceramics with base metal alloys of Ni-Cr, Cr-Co or titanium (Ti), have not yet fallen into disuse, due to their esthetic and functional benefits and lower cost [46]. However, the use of this type of restoration, together with poor hygiene practices and some systemic conditions of the patient, have caused changes in the periodontium, which accelerate its destruction and consequent dental mobility and loss of restored dental organ [177]. After some years of the patients having restoration in the mouth, this leads to the extraction and enlargement of the prosthesis, leaving many of them partially edentulous, having to use movable prosthesis that are cumbersome and uncomfortable, or having to undergo the placement of dental implants, with all the implications that this entails in terms of cost and invasion of the tissues. Therefore, in order to make prosthetic restorations a kinder local factor to the periodontium, new alternatives of esthetic and biocompatible materials such as monolithic zirconia have been sought [48] which produce fewer changes in the subgingival microbiota and therefore an increase in cytokines and other proinflammatory and destructive biomarkers, which will bring about periodontal deterioration [178].

The future search for better prosthetic biomaterials that cause fewer changes in the composition of the subgingival microbiota and biomarkers of inflammation and destruction of the periodontium should be aimed at:

1. The search for restorative materials or alloys with a composition as similar as possible to dental structures, which not only provoke a lower inflammatory response, but also favor periodontal health, i.e., materials that can permanently release ions that are bacteriostatic or selective bactericides.
2. The search for better luting materials that, if they spill into the periodontium, are not a retentive factor for bacteria, that can degrade easily in the oral environment, but at the same time that do not degrade inside the restoration.
3. Achieving an optimal marginal seal of the restorations, as close as possible to the natural cement-enamel bond, which will improve as the CAD/CAM systems for intraoral and extraoral scanning for the impression and fabrication of the restorations become more and more precise.

We must seek preservation and not mutilation, and although today implantology and implantation biomaterials are being revolutionized, there is nothing like natural teeth and the preservation of healthy periodontium. If prosthetic restorations are necessary, the biomaterials should be as close as possible to natural teeth, which do not cause undesirable inflammatory reactions, without neglecting the esthetics and masticatory function, which are important for the overall health of the individual.

## 9. Materials and Methods

The present study is a narrative review that included scientific articles, from which the most relevant information was summarized through a critical compilation of the information.

The information review was based on four different electronic databases involving PubMed/Medline, Science Direct, Scopus and Google Scholar. A combination of related keywords such as “prosthetic biomaterials”, “fixed prosthesis”, “periodontal health”, “subgingival microbiota”, “periodontal biomarkers” and “gingival crevicular fluid” were used. Relevant full-text articles published between 2000 and 2022 in dental journals were reviewed, including cross-sectional, longitudinal, case–control, randomized in vitro studies, and systematic and narrative reviews.

## 10. Conclusions

Metal-free ceramic prostheses induce a lower inflammatory response regardless of the fabrication method; however, the use of CAD/CAM systems is recommended for their fabrication. In addition, metal-ceramic prostheses induce changes in the composition of the subgingival microbiota producing a more dysbiotic biofilm with a higher prevalence of periodontopathogenic bacteria.

**Figure 3:**
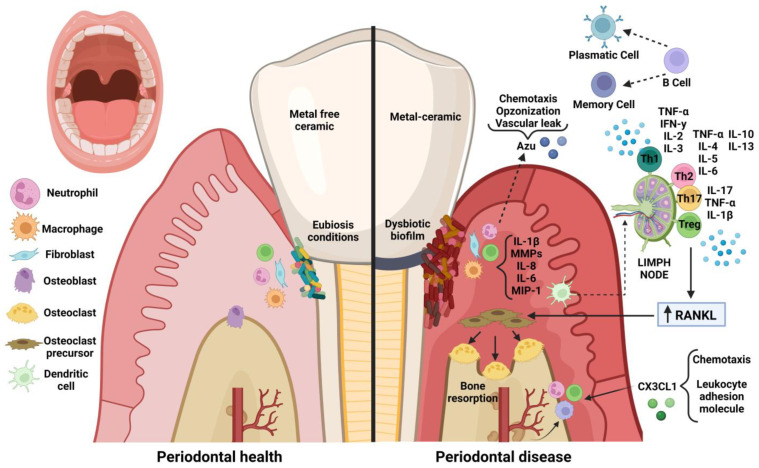
Comparison of periodontal immune response in the presence of metal-free ceramic and porcelain-metal prosthetic restorations. Azu: azurocidin; IL-1β: interleukin 1 beta; MMPs: matrix metalloproteases; IL-8: interleukin 8; IL-6: interleukin 6; MIP-1: macrophage inflammatory protein 1; CX3CL1: fractalkine; RANKL: receptor activator of nuclear factor κB ligand; Treg: regulatory T cells; Th17: T helper type 17 cells; Th2: T helper type 2 cells; Th1: T helper type 1 cells; TNF-α: tumor necrosis factor alpha; IL-17: interleukin 17; IL-4: interleukin 4; IL-5: interleukin 5; IL-10: interleukin 10; IL-13: interleukin 13; IL-2: interleukin 2;IL-3: interleukin 3; IFN-γ: interferon-gamma. www.biorender.com (accessed on 9 December 2022).

In response, neutrophils release Azurocidin which has a potent chemoattractant effect, induces vascular leakage and contributes to bacterial elimination by opsonizing bacteria, which facilitates recognition and uptake by phagocytes. Other cells such as gingival fibroblasts release chemokines such as fractalkine (CX3CL1) which has a dual function as a chemoattractant and cell adhesion molecule, which further contributes to leukocyte migration to fight infection. Moreover, keratinocytes, macrophages and lymphocytes release proinflammatory cytokines such as TNF-α and IL-1B that induce the expression of RANKL, which increases osteoclastogenesis and matrix metalloproteases (MMPs) that contribute to the degradation of the extracellular matrix. Dendritic cells recognize antigens and travel to lymph nodes to present the antigen to T lymphocytes that differentiate into subtypes depending on the antigen and cytokine environment. T cells induce cytokine secretion to regulate the immune response. Finally, B cells produce antibodies or become memory cells and contribute to the adaptive immune response (Figure 3) [179,180].

Some prosthetic factors such as poor marginal and internal fit, placement of very deep margins with invasion of the biological thickness, a very thin periodontal phenotype and poor oral hygiene could contribute to the pathogenesis of periodontal disease.

Thus, we propose the use of zirconia prosthetic restorations, as a very promising biomaterial with less negative effects on the periodontal condition of patients wearing fixed prostheses; however, studies on their effects on the subgingival microbiota, inflammatory mediators and the supporting tissues of the teeth supporting these prostheses are still needed.

Regarding AZU and CX3CL1, these molecules have been strongly related to periodontal disease; however, no studies have been carried out associating them with the subgingival microbiota or as part of the effects produced in patients with prosthetic restorations.

## Figures and Tables

**Table 1:** Studies evaluating the effects of different prosthetic restorative materials on biomarkers of inflammation in oral fluids.

Prosthetic Restorative Material	Biomarkers	Oral Fluid	Measurement Method	Biomarker Concentration and Main Findings	Reference
Metal-porcelain crowns with Cr-Co based alloy Zirconium dioxide crowns	CRP TNF-α YKL-40 Resistin AST ALP	GCF Serum	ELISA	CRP and TNF-α levels increased after placement of metal-porcelain prostheses compared with zirconia prostheses Metal-porcelain: YKL-40:56.32 ± 10.12 pg/mL. Resistin: 8.36 ± 2.01 pg/mL AST: 3.55 ± 1.01 pg/mL ALP: 3.55 ± 0.88 pg/mL Zirconium: YKL-40: 42.35 ± 9.65 pg/mL Resistin: 5.24 ± 1.65 pg/mL AST: 3.01 ± 0.80 pg/mL ALP: 3.11 ± 0.60 pg/mL	[26]
Ceramic Lumineers	IL-6 TNF-α	GCF	Luminex	IL-6 Baseline: 5.4 ± 3.6 pg/mL Week 4: 15.6 ± 8.2 pg/mL Week 12: 7.8 ± 6.2 pg/mL Week 24: 7.4 ± 5.2 pg/mL TNF-α Baseline: 13.7 ± 5.8 pg/mL Week 4: 65.3 ± 16.2 pg/mL Week 12: 25 ± 10.2 pg/mL Week 24: 21.3 ± 7.6 pg/mL	[27]
Temporary polymethylmethacrylate crowns Fixed zirconia crown	IL-1β	GCF	ELISA	IL-1β Group 1(Before temporary crown cementation): 13,587 pg/mL. Group 2 (2 weeks after temporary crown placement and before fixed crown placement): 9602 pg/mL. Group 3 (2 weeks after fixed crown placement): 6293 pg/mL	[28]
Porcelain Metal Crowns: Ceramic surface and metal surface Zirconia	IL-1β	GCF	ELISA	IL-1β Baseline: Ceramic:109.63 ± 14.49 pg/mL. Metal: 135.29 ± 18.63 pg/mL Zirconia: 86.57 ± 12.52 pg/mL 45 days: Ceramic: 106.80 ± 13.17 pg/mL Metal: 133.54 ± 18.89 pg/mL Zirconia: 87.54 ± 11.10 pg/mL 90 days: Ceramic: 102.25 ± 13.21 pg/mL Metal: 141.98 ± 27.72 pg/mL Zirconia: 79.88 ± 13.66 pg/mL	[29]
Stainless steel crowns	MIP-1 α MIP-1 β	GCF	ELISA	MIP-1 α: 682.55 ± 59.97 pg/mL MIP-1 β: 884.35 ±125.46 pg/mL	[30]
Lithium disilicate veneers Zirconia veneers Zirconia crowns	IL-1β IL-1ra aMMP-8	GCF	ELISA	Lithium disilicate veneers: IL-1β: 68.05 ± 50.80 pg/mL IL-1ra: 35.35 ± 2.355 pg/mL aMMP-8: 32.51 ± 33.08 pg/mL Zirconia veneers: IL-1β: 55.77 ± 37.33 pg/mL IL-1ra: 36.78 ± 20.87 pg/mL aMMP-8: 16.39 ± 10.10 pg/mL Zirconia crowns: IL-1β: 57.76 ± 61.10 pg/mL IL-1ra: 24.15 ± 21.67 pg/mL aMMP-8: 35.62 ± 35.60 pg/mL	[31]
Porcelain-metal crowns: Metal surface and ceramic surface Composite restorations Amalgam restorations	Substance P Neurokinin A Calcitonin gene-related peptide IL-1 α IL-1β PGE2	GCF	ELISA	Surface metal: Substance P: 3.85 (3.5–4.26) pg/mL Neurokinin A: 9.55 (0.04–10.3) pg/mL Calcitonin gene-related peptide: 5.71 (5.10–6.35) pg/mL IL-1 α: 0.68 (0.36–1.59) pg/mL IL-1β: 0.93 (0.64–1.09) pg/mL PGE2: 0.65 (0.56–0.87) pg/mL Ceramic surface: Substance P: 7.36 (3.69–13.49) pg/mL Neurokinin A: 9.61 (9.02–10.41) pg/mL Calcitonin gene-related peptide: 5.76 (5.10–6.25) pg/mL IL-1 α: 0.61 (0.10–2.12) pg/mL IL-1β: 0.81 (0.63–1.11) pg/mL PGE2: 0.63 (0.59–0.79) pg/mL	[32]
Full-coverage definitive restorations with different levels of crown margin placement	IL-1 α IL-8	GCF	ELISA	Supragingival margin: IL-1 α: 53.8 ± 9.7 pg/mL IL-8: 49.9 ± 9.7 pg/mL Equigingival margin: IL-1 α: 110.5 ± 23.3 pg/mL IL-8: 131.4 ± 27.5 pg/mL	[33]
Ceramic metal crowns With Ni-Cr alloy	IL-8 IL-6	GCF	ELISA	IL-8 Before restoration: 76.03 ± 31.49 pg/mL 1 week after: 79.13 ± 29.01 pg/mL 3 months after: 88.50 ± 30.46 pg/mL 6 months after: 82.87 ± 31.05 pg/mL IL-6 Before restoration: 265.97 ± 13.35 pg/mL 1 week after: 291.62 ± 17.75 pg/mL 3 months after: 311.34 ± 12.80 pg/mL 6 months after: 317 ± 14.45 pg/mL	[34]
Metal and metal-ceramic prosthetic restorations with Cr-Co and Ni-Cr alloys	MMP-2 MMP-8 MMP-9 IL-1β IL-6 TNF-α TIMP-1 TIMP-2	GCF	ELISA	Patients with prosthetic restorations and periodontitis have increased levels of TNF- α, MMP-8, IL-1β and IL-6 compared to patients without prosthetic restorations	[35]
Metal ceramic crowns Divided into healthy, gingivitis and periodontitis affected sites	IL-1β IL-6 TNF-α	GCF	ELISA	Sites affected with gingivitis and periodontitis were associated with significantly increased secretion of inflammatory cytokines in FCG compared to healthy sites	[36]
Crowns with different levels of placement of their margins	IL-1β MMP-2	GCF	ELISA	L-1β Before nonsurgical therapy: Supragingival margins: 49.6 pg/mL Gingival margins: 74.5 pg/mL Subgingival margins: 101.6 pg/mL After non-surgical therapy: Supragingival margins: 17.3 pg/mL Gingival margins: 65.7 pg/mL Subgingival margins: 57.7 pg/mL MMP-2 values were not detectable, because they are below the detection threshold of this test.	[37]
Abutment teeth supporting a fixed metal-ceramic partial denture	IL-6 IL-8	GCF	ELISA	IL-6 Initial values: 0.86 ± 1.21 pg/mL 1 month later: 0.99 ± 1.30 pg/mL 3 months later: 0.57 ± 0.68 pg/mL IL-8 Initial values: 0.93 ± 0.81 pg/mL 1 month after: 0.91 ± 0.65 pg/mL 3 months after: 0.50 ± 0.34 pg/ml	[38]
Galvanic-ceramic crowns Metal-ceramic crowns	IgG	GCF	ELISA	IgG Baseline: 542.28 ± 1078.54 pg/mL 12 months after: 264.61 ± 532.24 pg/mL 24 months later: 390.41 ± 908.62 pg/mL	[39]
Abutment teeth supporting a removable partial denture made of metal (Cr-Co alloy) and acrylic resin.	IL-1β	GCF	ELISA	IL-1β Baseline: 133.1 ± 52.1 pg/mL 9 months later: 122.7 ± 30.1 pg/mL	[40]
Ceramic metal crowns: Group 1: Cr-Ni-M alloy ceramics. Group 2: Ceramics Group 3: Au-Pt-In alloyed ceramics	IL-1β	GCF	ELISA	IL-1β Group 1: 95.31 ± 29.19 pg/mL Group 2: 93.63 ± 45.06 pg/mL Group 3: 103.4 ± 54.34 pg/mL	[41]

GCF: gingival crevicular fluid; Ni-Cr: nickel-chromium; Cr-Co: chromium-cobalt; Au-Pt: gold-platinum; Cr-Ni-M: chromium-nickel-molybdenum; TNF-α: tumor necrosis factor alpha; CRP: C-reactive protein; YKL-40: chitinase 3-like protein 1; AST: aspartate aminotransferase; ALP: alkaline phosphatase; IL-6: interleukin 6; IL-8: interleukin 8; IL-1 α: interleukin 1 alpha; IL-1β: interleukin 1 beta; IL-1ra: interleukin 1 receptor antagonist; MIP-1α: macrophage inflammatory protein 1 alpha; MIP-1β: macrophage inflammatory protein 1 beta; PGE2: prostaglandin E2; MMP-2: matrix metalloprotease 2; MMP-8: matrix metalloprotease 8; aMMP-8: matrix metalloprotease 8 in its active form; MMP-9: matrix metalloprotease 9; TIMP-1: tissue inhibitor of metalloproteases 1; TIMP-2: tissue inhibitor of metalloproteases 2; IgG: immunoglobulin G.

## Data Availability

Not applicable.
